# Functional MRI in Awake Unrestrained Dogs

**DOI:** 10.1371/journal.pone.0038027

**Published:** 2012-05-11

**Authors:** Gregory S. Berns, Andrew M. Brooks, Mark Spivak

**Affiliations:** 1 Center for Neuropolicy, Emory University, Atlanta, Georgia, United States of America; 2 Comprehensive Pet Therapy, Atlanta, Georgia, United States of America; University Zürich, Switzerland

## Abstract

Because of dogs' prolonged evolution with humans, many of the canine cognitive skills are thought to represent a selection of traits that make dogs particularly sensitive to human cues. But how does the dog mind actually work? To develop a methodology to answer this question, we trained two dogs to remain motionless for the duration required to collect quality fMRI images by using positive reinforcement without sedation or physical restraints. The task was designed to determine which brain circuits differentially respond to human hand signals denoting the presence or absence of a food reward. Head motion within trials was less than 1 mm. Consistent with prior reinforcement learning literature, we observed caudate activation in both dogs in response to the hand signal denoting reward versus no-reward.

## Introduction

As the oldest domesticated species, with estimates ranging from 9,000–30,000 years BCE, the minds of dogs inevitably have been shaped by millennia of contact with humans [1], [2]. As a result of this physical and social evolution, dogs, more than any other species, have acquired the ability to understand and communicate with humans. A resurgence of research in canine cognition has revealed the range (and variability) of skills such as following pointing and gaze cues [3], [4], [5], fast mapping of novel words [6], and the conjecture that dogs have emotions [7]. Although the growing list of canine cognitive skills is impressive, how does the dog mind actually work? We are left to infer canine brain function from behavior and ultimately guess at the inner workings of the dog brain. However, the widespread use of functional magnetic resonance imaging (fMRI) to study brain function in both humans and non-human primates has paved the way for potentially understanding how the dog brain works. Here, we report the development of behavioral and technical methods to acquire fMRI data in fully awake, unrestrained dogs.

The main challenge of fMRI in dogs comes from subject motion. Historically, the usual approach has been to either anesthetize the animal [8], [9] or, as in rats and monkeys, immobilize them [10], [11], [12], [13], [14], [15]. Clearly, if we wish to understand canine cognition, anesthesia is not an option. Immobilization is technically possible, although ethically objectionable for a dog, and, as we show, unnecessary to acquire useful fMRI data. Instead, because dogs so readily follow human commands, they can be trained to go into an MRI scanner and hold their head still enough for fMRI studies without any restraint. Moreover, they will do this happily with nothing more than positive reinforcement.

Because of their prolonged evolution with humans, many of the canine cognitive skills are thought to represent a selection of traits that make dogs particularly sensitive to human cues [16]. For this reason, we selected a simple discrimination task with two human hand signals for initial study with canine fMRI. Although there is growing evidence that dogs do not need to be conditioned to learn human hand signals, for this first experiment we chose to associate the hand signals with primary rewards to provide a linkage with comparable imaging experiments in both humans and monkeys and to maximize the chance of observing a significant brain response. Importantly, the reward-prediction error hypothesis of the dopamine system provides a concrete prediction of activity in the ventral caudate of the dog. The task was designed to determine which brain circuits differentially respond to hand signals denoting the presence or absence of a food reward. Based on the reinforcement learning literature, we hypothesized that the transfer of reward association to a hand signal would manifest in the ventral striatum [17], [18], [19], [20], [21].

## Results

Subjects were two spayed, female, domesticated dogs. Callie was a 2 year-old feist of indeterminate pedigree, who had been adopted from a local shelter at the age of 9 months and weighed 12 kg. Apart from basic obedience, she had no specialized training. McKenzie was a 3 year-old border collie and was already well-trained in agility competition and weighed 16 kg. Training and handling for the following procedures were performed by each dog's owner under the supervision of a professional trainer. This study was performed in strict accordance with the recommendations in the Guide for the Care and Use of Laboratory Animals of the National Institutes of Health. All procedures were approved by the Institutional Animal Care and Use Committee of Emory University (Protocol Number: DAR-2001274-120814).

Three fMRI scanning sessions were performed over a period of 6 weeks. Callie participated in all sessions, while McKenzie participated in the last two. The goal of the first session was to familiarize the Callie with the scanner environment and determine the feasibility of acquiring both structural and fMRI data. The goal of the second session was to optimize the scan parameters and to obtain enough fMRI data to evaluate its quality for movement-related artifacts. It was observed that the onset of each imaging sequence tended to startle the dogs, causing them to move or exit the scanner. This was effectively mitigated in the final session by playing recordings of the scanner noise through the intercom while the dog got settled into the chin rest. The preceding protocol encouraged habituation to the scanner noise and eliminated startle reactions. In the third and final session, the onset was not startling and the dogs didn't move severely when the actual sequence started. This approach allowed us to obtain functional runs long enough for fMRI analysis as well as a high quality structural image.

**Figure 1:**
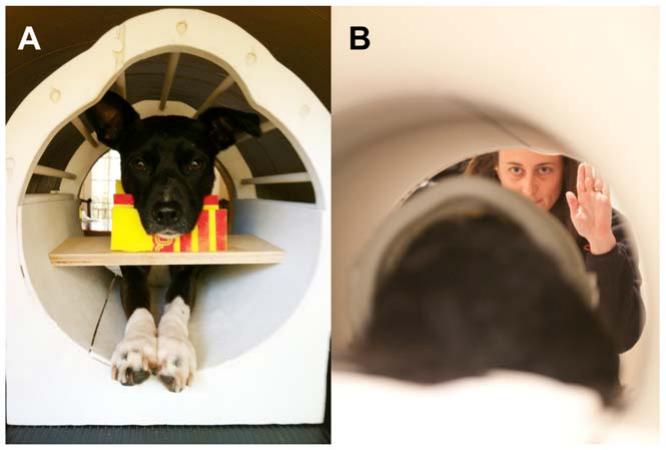
Training and task for dogs in the MRI scanner. (A) Callie in the training apparatus, which consisted of a replica of the head coil inside a tube of the approximate diameter of the MRI bore. Consistent positioning of the head was achieved by training the dog to place her head in a chin rest molded to the lower jaw from mid-snout to behind the mandible. The chin rest was affixed to a wood shelf that spanned the head coil but allowed enough space for the paws underneath. No restraints were used. The training procedure gradually shaped the desired behavior of placing the head in the rest and not moving through positive reinforcement only. Dogs were free to exit the apparatus at any time. (B) McKenzie inside the real head coil in the MRI. Her handler is giving a hand signal that denotes upcoming “reward.” We used a simple instrumental conditioning task in which the required behavior was to place the head on the chin rest and not move. After a variable interval of approximately 5 s, a hand signal was given that indicated whether a reward would be delivered. The dog had to continue holding still during this period to get the reward. The left hand up indicated a hot dog reward, while both hands pointing toward each other horizontally indicated no-reward. The hand signals were maintained for approximately 10 s. Reward-trials ended by the handler reaching in with the food to the dog. Person in the photograph has given written informed consent for publication.

For the final scanning session, we used a simple instrumental conditioning task in which the required behavior was to place the head on the chin rest and not move (Fig. 1). After a variable interval of approximately 5 s, a hand signal was given that indicated the presence or absence of a food reward that would be received. The left hand up indicated a hot dog reward, while both hands pointing toward each other horizontally indicated no reward. The hand signals were chosen to be easily distinguishable and were maintained for approximately 10 s. The dog had to continue holding still during this period. Dogs had been amply trained on these hand signals in the simulator prior to the final scan session. Because the dogs had been trained to go into the head coil in a “sphinx” position (Fig. 1), the handler gave the hand signals from the head end of the scanner, facing the dog. Trial types were approximately random and alternating (but not predictably) such that we had an approximately equal number of both trial types.

FMRI data were acquired on a Siemens 3 T Trio. We used a single-channel transmit-receive head coil because of its large size and ability to accommodate the dog in the sphinx position. The chin rest was constructed to fit inside the coil. First, a single sagittal plane image was acquired as a localizer, which lasted 3 s. For functional scans, we used single-shot echo-planar imaging (EPI) to acquire volumes of 28 sequential 3 mm slices with a 10% gap (TE = 28 ms, TR = 1610 ms, flip angle = 70°, 64×64 matrix, FOV = 192 mm). Slices were oriented dorsally to the dog's brain (coronal to the magnet because the dog was positioned 90° from the usual human orientation) with the phase-encoding direction left-to-right (Fig. S1). For each dog, two runs of 190 volumes were acquired, each lasting 5 minutes, during which the reward/no-reward task was performed. For Callie, this yielded a total of 19 reward trials and 20 no-reward, and 16 reward and 11 no-reward trials for McKenzie (but of longer duration). After the functional runs, a T2-weighted structural image was acquired with a turbo spin-echo sequence (30 3 mm slices, TR = 3710, TE = 8.3, 26 echo trains), which lasted 24 s. This sequence was optimized to yield good contrast between grey and white matter in the fastest time possible. The dog was required to hold still for the entire 24 s, after which she was rewarded.

**Figure 2:**
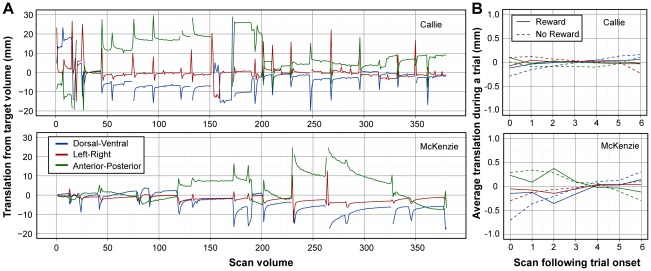
Motion during canine fMRI. (A) Timeseries of translations required to correct for motion during the scan sessions. Volume 32 was the target for Callie, and volume 1 was the target for McKenzie. The plots therefore represent the total movement from the target volume. The spikes and breaks occurred when the dog moved its head out of the field of view, which typically happened following a reward. The volumes with artifacts were excluded from further analysis, leaving 62% of the volumes for Callie and 58% for McKenzie. Although the dogs did not place their heads back in exactly the same position, once they did, very little motion was observed. McKenzie exhibited a slow anterior-posterior drift during the second run, but this was sufficiently slow as to not cause movement artifacts during trials. (B) Average motion during a trial, separated by reward and no-reward conditions and after exclusion of volumes with artifacts. Scan volumes are 1610 ms apart. Notably, within-trial motion was less than 1 mm in all directions for both dogs, and no difference between the reward and no-reward conditions was observed.

Data were processed with AFNI. Because the dogs exited the scanner between runs, head positioning was slightly different. Using fiducial markers on the brain, we roughly aligned the second run to the first. Next, we used a two-pass motion correction to complete the alignment and generate measurements of movement within each run (Fig. 2A). Because many trials ended with a food reward, the dog moved her head while consuming the treat, but once consumed, she placed her head back in the chin rest. Movement and loss-of-shim artifacts were expected during this period. A large field of view guaranteed that the entire brain was captured regardless of the exact trial-to-trial position. Activation time series were examined and censored for artifacts through a multistep process [22]. Volumes with obvious movement were excluded and the remaining volumes used to calculate percent signal change on a voxelwise basis. We then excluded any volume in which the signal, averaged over the whole brain, changed by more than 1% from the previous scan. Finally, the sequence of scans was examined in a movie loop and any remaining scans that exhibited sudden movements were excluded. This resulted in the retention of 236 out 380 volumes for Callie (62%) and 222 volumes for McKenzie (58%). Although the inter-trial movements were large compared to humans, once set in the chin rest, the dogs were comparable, if not better, than humans. The average total translation within each trial was less than 1 mm (Fig. 2B). The results of motion correction were checked by scrolling through time in AFNI to confirm that the brain remained in the same position throughout the retained scans (see Movies S2 and S3). Despite the fact that the dogs went in and out of the field, after motion correction the brain was observed to stay in the same position within a voxel. To account for any remaining variance due to misalignment and to improve the signal to noise ratio, scans were then smoothed with a 6 mm gaussian kernel.

Key events of each trial were marked by an observer with button presses and logged to a computer capturing scanner pulses. These events were used to formulate a GLM for analysis of the fMRI data: 1) reward hand signal; 2) no-reward hand signal; 3) and reward. The hand signals were specified as variable duration events, while the reward was specified as an impulse. All events were convolved with a standard hemodynamic response function. The design matrix also included constants and linear drifts for each run, and the six motion parameters. A censor file specified the volumes to be excluded from the regression.

**Figure 3:**
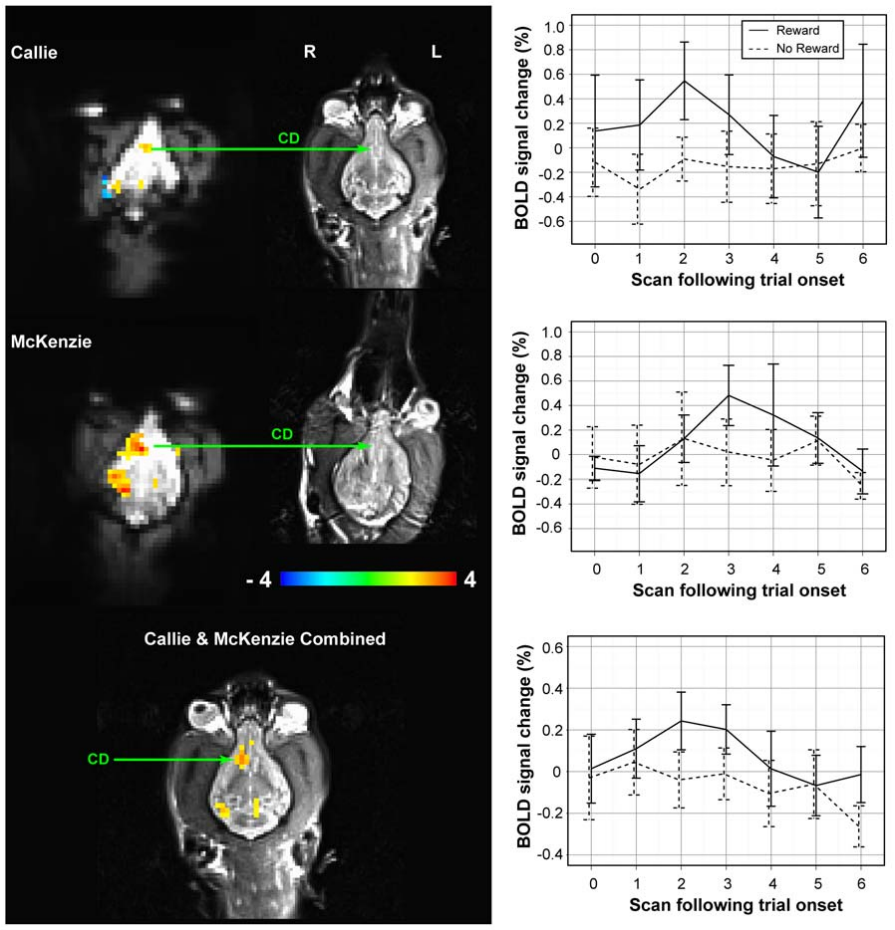
The caudate is significantly more active to the “reward” hand signal compared to the “no-reward” hand signal. The same region of activation was observed in both dogs and is identified as the right caudate as indicated on the corresponding slice of each dog's structural image (*CD*). The structural image has been uniformly scaled to match the size of the brain of the functional images. The underlay of the functional map is the mean of the non-excluded functional images. McKenzie was rotated slightly out of plane, but this was a consistent position in both functional and structural scans. The significance of the peak voxel in this cluster was p<0.01 in Callie and p<0.001 in McKenzie (colorbar indicates t-values and maps are thresholded at p<0.05 to show full spatial extent). The time series of activation was extracted for the cluster (9 voxels in Callie, and 18 voxels in McKenzie after restricting spatial extent with p<0.01), and after adjusting for the other effects in the design matrix (including motion), the average trial response is seen to match a typical hemodynamic response function, which is significantly greater for the “reward” signal than the “no-reward” signal (error bars are +/− 1 s.e.) *Bottom*: statistical map of the combined model with both dogs, co-registered and overlaid on Callie's structural scan. Activation of the caudate cluster (*CD*) was significant at p<0.05 after correcting for FDR over the search volume of the ventral brain from olfactory bulb to internal capsule (p<0.01 height and cluster extent>6). Averaged over both dogs, the timecourse of activation in the caudate showed a distinct response to the reward hand signal which differentiates from the no-reward signal (*lower right*). Scan volumes are 1610 ms apart, indicating a peak in the response 3–5 s after the onset of the reward hand signal.

Because of the weight of evidence implicating the ventral striatum in reward-prediction error learning, we focused our analysis on the head of the caudate. In the dog, the caudate is located ventral to the genu of the corpus callosum, between the olfactory peduncle and anterior limb of the internal capsule [23], [24], [25]. The latter is easily identified on T2-weighted images as two dark diagonal lines (Fig. S2, S3, S4, S5). The contrast of reward versus no-reward hand signals revealed a significant cluster of activation in the region of the right caudate of both dogs (Fig. 3). With the entire extent of activation displayed in all slices, and referenced to the corresponding slices of the T2 image, it is clear that these clusters are very close to, if not exactly on, the caudate (Figs. S2, S3, S4, S5). Although the statistical significance of the caudate cluster was modest in each dog individually (p<0.01 in Callie, and p<0.001 in McKenzie), the observation of the same location in the same condition in both dogs, and in the hypothesized region, strongly suggests that these were not spurious findings. The average trial responses to the hand signals showed a distinct hemodynamic response to the reward signal but not the no-reward signal, which would be expected for the association of reward to one signal but not the other (Fig. 3). When the datasets of both dogs were combined by spatial warping, activation of the caudate cluster was significant at p<0.05 after correcting for FDR over the search volume of the ventral brain from olfactory bulb to internal capsule (p<0.01 height and cluster extent>6).

## Discussion

Based on the vast reinforcement learning literature, the observation of caudate activation to a hand signal associated with reward is not surprising. In fact, had this not been observed, one could rightfully question the feasibility of canine fMRI. The reward prediction error hypothesis of dopamine function suggests that dopamine is released in response to unexpected events that signal future reward [17], [18], [26], [27]. Although not directly measuring dopamine, many fMRI studies have found that the BOLD signal in the ventral striatum also follows this pattern of activation [19], [20], [28], [29], [30], [31], [32]. Thus, it is likely that the caudate signal we observed represents a positive reward prediction to the dog. We assume that this is because of the trained association to a food reward; however, it is also possible that some component of social reward contributes to the response. Future studies could separate these potential components by implementing cues from humans and inanimate sources (e.g. lights), for example. Moreover, the stronger response observed in McKenzie may reflect the extensive agility training she had undergone with her handler, in effect, making her more attuned to hand signals than Callie. Future studies may reveal the sources of such heterogeneity including training, temperament, and reward modality.

We associated the hand signals with primary reward in order to maximize the chance of detecting activation in the dog's reward system. With only two dogs, the odds of detecting such activation were quite low, but the observation of ventral caudate activation in both dogs clearly shows that canine fMRI is not only possible, but paves the way for studying canine social cognition. Because there was no associated behavior for the hand signals, we can't say how many trials it took for them to learn the association, but itt was over a period of weeks with daily 10 minute sessions. Future studies can now determine, for example, whether the hand signals were intrinsically rewarding because they came from the dogs' owners (e.g. a social reward), or whether they were rewarding only because of the association with food.

The possibility of future canine fMRI must be tempered with the acknowledgement that dogs will do almost anything humans ask of them, and this makes them particularly vulnerable to exploitation. In the design and implementation of this study, we adopted a set of principles that places the dogs' welfare above all else, and which we hope will provide ethical guidelines for future work in this area [7]. First, no harm must occur to the dogs. With MRI, the main concern is for the dogs' hearing, which is more sensitive than humans'. Considerable effort was spent fitting and training the dogs to wear ear muffs and head wraps that mitigated the effects of the scanner noise. Second, the dogs should not be restrained. Although it is technically possible to implement a wide range of restraints, from harnesses to implanted fixation devices, we believe this violates a basic principle of self-determination that is normally reserved for humans, but in this case should be extended to dogs: theyshould be free to exit the scanner at all times. Similarly, this means that purpose-bred laboratory dogs should not be used as they have no choice. Third, positive reinforcement should be used whenever possible. Although we can imagine experiments in which one would like to know the differential effects of positive reinforcement versus punishment, we favor positive reinforcement for ethical reasons. The use of punishment should be carefully weighed against the alternatives, especially since the animal training literature does not indicate that punishment leads to more effective learning than positive methods.

To our knowledge, this is the first demonstration of either MRI or fMRI in completely awake, unrestrained dogs. The quality of the structural images alone, especially of Callie, demonstrate that dogs can hold as still as humans for periods up to 24 s – long enough for a wide variety of functional studies and veterinary applications. Future technical advancements, including the use of parallel imaging and sensors for movement, should allow for even higher quality data by shortening the scan times and more easily identifying movement artifacts. Although we chose a simple instrumental conditioning task to demonstrate the feasibility of canine fMRI, a wide variety of future studies is now possible. Dogs have had a prolonged evolution with humans, and they are uniquely attuned to our behaviors. For example, one might reasonably ask to what extent the dog mentalizes the minds of humans. Dogs are intensely visual and pay attention to our facial expressions and where we look and point. How do they represent these actions? How do dogs distinguish humans, and is it by vision or smell? Is human language processed as arbitrary sounds, or do dogs have neural structures that respond in a deeper manner to language? What is the difference between how dogs represent humans and other dogs or animals? The questions are endless. And while the study of the canine mind is fascinating for its own sake, it also provides a unique mirror into the human mind. Because humans, in effect, created dogs through domestication, the canine mind reflects back to us how we see ourselves through the eyes, ears, and noses of another species.

## Materials and Methods

### Training Procedure

Although this research does not require the use of lab animals (e.g. purpose-bred research dogs), not all dogs are appropriate for this type of research. We recruited dogs who were already well-socialized with humans, specifically pet dogs and their owners. Prior to scanning, the dogs were evaluated for appropriate temperament. Ideal characteristics included calmness, evidence of curiosity, not fearful of strangers or other dogs, calmness when transitioning to novel environments, not afraid of loud noises, not afraid of heights, the ability to remain relaxed in an enclosed environment, and most importantly, evidence of motivational drive during training. This last characteristic was important given the sedentary nature of the task on which they were to be trained. The dogs underwent extensive behavioral training to acclimate them to the MRI environment. To do this, we constructed two MRI simulators, which consisted of exact replicas of the head coil, a tube of approximately the same dimensions of the inner bore of the MRI, a patient table within the tube, all of which was placed on a collapsible table at the approximate height of the scanner table. Recordings of the scanner sequences were played through a P.A. system aimed at the simulator. Sound pressure levels were verified with a handheld decibel meter and confirmed at 95–96 dB. The simulators were located at the owners' homes or the training facility to allow for daily training and to let the dogs become comfortable with the apparatus in a familiar environment.

Only positive reinforcement, in combination with behavioral shaping, conditioning and chaining, were used in the training process, which took place over a period of 2 months. First, dogs were trained to place their head and paws in the head coil. Next, they were trained to place their chin on a foam bar placed horizontally across the head coil and hold this position until a release signal. The length of the hold was gradually increased up to 30 s. The chin rest was subsequently modified to a custom fit based on the chin shape. When the dogs were able to do this consistently with no discernible head motion, they were next trained to do this wearing canine ear muffs, which were initially introduced to the animals apart from the coil simulator. Concurrent with the initial sequences of the training, recordings of the scanner noise were introduced at low volume. Once the animal became conditioned at a low volume, the volume was gradually increased. After each dog reached a hold time of 30 s within the coil simulator, recordings of the scanner noise were introduced at low volume while the dog remained stationary in the coil. Once the dog demonstrated relaxed behavior, the volume was gradually increased. When the dogs were comfortable wearing the ear muffs in the head coil with the scanner noise of approximately 90 dB, they were then trained to go into the MRI tube which had been placed on the floor. This was not difficult, and subsequently, the simulated head coil was placed inside the tube. Finally, after the dog was consistently holding its head still in this configuration, the entire apparatus was raised on a table to the height of the actual scanner patient table. At this point, the dog was trained to walk up a set of steps into the tube and assume the correct position (see Movie S1).

### Data Acquisition and Analysis

#### Key event recording

Trial events were recorded by an observer via a four-button MRI-compatible button-box. A laptop running Matlab (MathWorks) and Cogent (FIL, University College London) was connected via serial port to the button box, and recorded both the button-box responses by the observer to the dogs, as well as scanner sequence pulses.

#### Functional data pre-processing

All functional data processing was completed using AFNI and its associated functions. DICOM images of the EPI runs were first converted to the AFNI BRIK format using the to3d command. Because the brain was in a slightly different location for each run, the second run was coarsely aligned to the first run based on five fiducial tags, which were easily identifiable in both runs: tip of the olfactory bulb, mid corpus callosum, the left and right edge of the brain (in the same slice as corpus callosum), center of the dorsal hindbrain, and the anterior temporal lobe. To do this, the 3dTagalign function was used.

After fiducial marker-based alignment, both runs were concatenated into a single BRIK file with 3dTcat. Motion correction was run on the concatenated volume via 3dvolreg, and implemented with a two-pass iterated linearized weighted least squares approach, where each volume was aligned to the first good volume of run 1 (volume 32 for Callie and volume 1 for McKenzie). The first pass used linear interpolation weighted by a mask of the brain, generated from the first run, to generate a crude alignment. The second pass used Fourier interpolation for finer alignment. Volumes which contained gross motion artifact, such as when the dog moved its head out of the RF coil, as well as volumes where the signal averaged across the brain changed by more than 1% from the previous scan were excluded from analyses using AFNI's built-in censor function. Remaining volumes were then smoothed with a 6 mm full-width-half-maximum Gaussian kernel to account for any remaining small misalignments with 3dmerge. Using 3dcalc, all voxels were converted to percent signal change by subtracting the mean of its time series and then dividing by its mean. Finally, the concatenated BRIK was split back into their respective runs with 3dTcat, for inclusion in the GLM and separate modeling of the runs.

Using 3dDeconvolve, the following events were used to formulate a GLM for analysis of the fMRI data: 1) reward hand signal; 2) no-reward hand signal; 3) and reward. The hand signals were specified as variable duration events, while the reward was specified as an impulse. The average duration of reward and no-reward events were within one second of each other (9.9 s & 8.9 s and 15.8 s & 16.4 s for reward & no-reward in Callie & McKenzie respectively). All events were convolved with a standard hemodynamic response function. The design matrix also included constants and linear drifts for each run, and the six motion parameters. A censor file specified the volumes to be excluded from the regression. The contrast of interest was between reward and no-reward hand signals. Because of the prior hypothesis regarding the caudate, we considered activations in this region significant at p<0.01 (although p<0.05 was used to visualize the full extent of activation). No inferences outside this region were made. The mean time series of activation was extracted from the region of the right caudate. To yield approximately the same size cluster in both dogs, a threshold of p<0.05 was used for Callie (9 voxels) and p<0.01 for McKenzie (18 voxels). Using Matlab, two adjusted time series were created for reward hand signal and no-reward hand signal by controlling for all of the effects other than the one of interest: constant, linear drift, 6 motion parameters, reward receipt, and the other hand signal. The average response for the two trial types was then calculated for the 6 volumes following the onset of the hand signal.

To create a combined activation map of the two dogs, McKenzie's mean motion-corrected functional image was aligned to Callie's mean motion-corrected functional image using fiducial tagging. This yielded a transformation matrix which was then applied to each of McKenzie's normalized, smoothed, and motion-corrected functional run images. Because of the small sample size (n = 2), a fixed-effects GLM model was run, in which a constant and linear drift term was included for each run and each subject. The same regressors for the subject-wise GLM described above were also included in the group model. The resultant statistical maps were co-registered to Callie's anatomical image for visualization and verification of the caudate activity.

Given the prior hypothesis about caudate activation, statistical inferences for the combined model were based on a masked region of the brain around the caudate. To do this, we created a mask (152 voxels) encompassing all regions inferior to the corpus callosum and anterior to the internal capsule and used Alpha Sim to calculate a mask FDR<0.05 (10,000 iterations). Smoothness estimates of the model residuals were calculated using 3dFWHMx. With a voxel-level threshold of *p*<0.01, AlphaSim yielded a cluster threshold of 6 voxels, such that the FDR within the masked region would be less than 0.05.

## Supporting Information

Figure S1 **Siemens 3 T Trio console screenshot, showing field-of-view (FOV) in Callie for both functional and structural scans.** The FOV was determined based on a localizer acquired prior to functional scan acquisition. Slices for the functional run were oriented dorsally to the dog's brain (similar to axial in humans). This was approximately coronal to the magnet because the dog was positioned 90° from the usual human orientation. The generous FOV, with extra slices dorsally and ventrally, allowed for different head positioning between trials.
(TIF)

Figure S2 **Labeled montage of Callie's T2-weighted structural image.** A T2-weighted structural image was acquired after the functional runs. The image was acquired using a turbo spin-echo sequence (30 3 mm slices, TR = 3710, TE = 8.3, 26 echo trains), which was optimized to yield contrast between gray and white matter in the fastest possible time. The red outline corresponds to the slice shown in Fig. 3, where the caudate shows greater activation to the reward hand signal versus no-reward hand signal. Primary and adjacent slices are labeled with the olfactory peduncle (OLF), cerebellum (CBL), caudate (CD), internal capsule (IC), and genu of the corpus callosum (CC).
(TIF)

Figure S3 **Labeled montage of Callie's mean motion-corrected EPI image.** EPI images were acquired using single-shot echo-planar imaging (28 3 mm slices, 10% gap, TE = 28 ms, TR = 1610 ms, flip angle = 70°, FOV = 192 mm). The mean image across runs was calculated by taking the average of all motion-corrected EPI volumes that did not exhibit significant motion artifact. This included obvious motion artifact related to withdrawal from the radiofrequency coil, and those volumes in which the average signal changed more than 1%. The red outline corresponds to the slice shown in Fig. 3, where the caudate shows greater activation to the reward hand signal versus no-reward hand signal. Primary and adjacent slices are labeled with easily distinguishable landmarks: the olfactory peduncle (OLF), internal capsule (IC), and genu of the corpus callosum (CC).
(TIF)

Figure S4 **Labeled montage of McKenzie's T2-weighted structural image.** A T2-weighted structural image was acquired after the functional runs. The image was acquired using a turbo spin-echo sequence (30 3 mm slices, TR = 3710, TE = 8.3, 26 echo trains), which was optimized to yield contrast between gray and white matter in the fastest possible time. The red outline corresponds to the slice shown in Fig. 3, where the caudate shows greater activation to the reward hand signal versus no-reward hand signal. Primary and adjacent slices are labeled with the olfactory peduncle (OLF), cerebellum (CBL), caudate (CD), internal capsule (IC), and genu of the corpus callosum (CC).
(TIF)

Figure S5 **Labeled montage of McKenzie's mean motion-corrected EPI image.** EPI images were acquired using single-shot echo-planar imaging (28 3 mm slices, 10% gap, TE = 28 ms, TR = 1610 ms, flip angle = 70°, FOV = 192 mm). A mean image across runs was calculated by taking the average of all motion-corrected EPI volumes that did not exhibit significant motion artifact. These included obvious motion artifact from head withdrawal from the coil, and those volumes in which the average signal changed more than 1%. The red outline corresponds to the slice shown in Fig. 3, where the caudate shows greater activation to the reward hand signal versus no-reward hand signal. Primary and adjacent slices are labeled with easily distinguishable landmarks: the olfactory peduncle (OLF), internal capsule (IC), and genu of the corpus callosum (CC).
(TIF)

Movie S1 Training video. This video shows how the dogs were trained to remain stationary in the MRI while wearing ear muffs. The video shows initial exposure to final behavior, which took place over a period of 2 months.
(MP4)

Movie S2 Video of raw fMRI scans for Callie after motion correction. Rapidly scrolling through the sequence of fMRI volumes shows that the brain is stationary within one voxel after motion correction has been performed and volumes with artifacts are excluded.
(MOV)

Movie S3 Video of raw fMRI scans for McKenzie after motion correction. Rapidly scrolling through the sequence of fMRI volumes shows that the brain is stationary within one voxel after motion correction has been performed and volumes with artifacts are excluded.
(MOV)

## References

[1] Shipman P (2011). The Animal Connection. A New Perspective on What Makes Us Human.

[2] Bradshaw J (2011). Dog Sense. How the New Science of Dog Behavior Can Make You a Better Friend.

[3] Hare B, Brown M, Williamson C, Tomasello M (2002). The domestication of social cognition in dogs.. Science.

[4] Hare B, Tomasello M (2005). Human-like social skills in dogs?. Trends Cogn Sci.

[5] Teglas E, Gergely A, Kupan K, Miklosi A, Topal J (2012). Dogs' gaze following is tuned to human communicative signals.. Current Biology.

[6] Kaminski J, Call J, Fischer J (2004). Word learning in a domestic dog: evidence for “fast mapping”.. Science.

[7] Bekoff M (2007). The Emotional Lives of Animals.

[8] Willis CKR, Quinn RP, McDonell WM, Gati JS, Parent J (2001). Functional MRI as a tool to assess vision in dogs: the optimal anesthetic.. Veterinary Ophthalmology.

[9] Aguirre GK, Komaromy AM, Cideciyan AV, Brainard DH, Aleman TS (2007). Canine and human visual cortex intact and responsive despite early retinal blindness from RPE65 mutation.. PLoS Medicine.

[10] Stefanacci L, Reber P, Costanza J, Wong E, Buxton R (1998). fMRI of monkey visual cortex.. Neuron.

[11] Logothetis NK, Pauls J, Augath M, Trinath T, Oeltermann A (2001). Neurophysiological investigation of the basis of the fMRI signal.. Nature.

[12] Leite FP, Tsao D, Vanduffel W, Fize D, Sasaki Y (2002). Repeated fMRI using iron oxide contrast agent in awake, behaving macaques at 3 Tesla.. Neuro Image.

[13] Keliris GA, Shmuel A, Ku S-P, Pfeuffer J, Oeltermann A (2007). Robust controlled functional MRI in alert monkeys at high magnetic field: effects of jaw and body movements.. Neuro Image.

[14] Chen G, Wang F, Dillenburger BC, Friedman RM, Chen LM (2012). Functional magnetic resonance imaging of awake monkeys: some approaches for improving imaging quality.. Magnetic Resonance Imaging.

[15] Kulkarni P, Stolberg T, Sullivan JM, Ferris CF (2012). Imaging evolutionarily conserved neural networks: preferential activation of the olfactory system by food-related odor.. Behavioural and Brain Research.

[16] Udell MAR, Dorey NR, Wynne CDL (2010). What did domestication do to dogs? A new account of dogs' sensitivity to human actions.. Biological Reviews.

[17] Schultz W, Apicella P, Scarnati E, Ljungberg T (1992). Neuronal activity in monkey ventral striatum related to the expectation of reward.. Journal of Neuroscience.

[18] Schultz W, Dayan P, Montague PR (1997). A neural substrate of prediction and reward.. Science.

[19] Berns GS, McClure SM, Pagnoni G, Montague PR (2001). Predictability modulates human brain response to reward.. Journal of Neuroscience.

[20] O'Doherty JP, Deichmann R, Critchley HD, Dolan RJ (2002). Neural responses during anticipation of a primary taste reward.. Neuron.

[21] Balleine BW, Delgado MR, Hikosaka O (2007). The role of the dorsal striatum in reward and decision-making.. Journal of Neuroscience.

[22] Stoewer S, Goense J, Keliris GA, Bartels A, Logothetis NK (2012). An analysis approach for high-field fMRI data from awake non-human primates.. PLoS One.

[23] Assheuer J, Sager M (1997). MRI and CT Atlas of the Dog.

[24] Saveraid TC, Fletcher TF (2009). Canine Head MRI Atlases.

[25] Palazzi X (2011). The Beagle Brain in Stereotaxic Coordinates.

[26] Sutton RS, Barto AG, Gabriel M, Moore J (1990). Time-derivative models of pavlovian reinforcement.. Learning and Computational Neuroscience: Foundations of Adaptive Networks.

[27] Wightman RM, Robinson DL (2002). Transient changes in mesolimbic dopamine and their association with ‘reward’.. Journal of Neurochemistry.

[28] O'Doherty J, Dayan P, Schultz J, Deichmann R, Friston K (2004). Dissociable roles of ventral and dorsal striatum in instrumental conditioning.. Science.

[29] Delgado MR, Nystrom LE, Fissel C, Noll DC, Fiez JA (2000). Tracking the hemodynamic responses to reward and punishment in the striatum.. Journal of Neurophysiology.

[30] Hampton AN, O'Doherty JP (2007). Decoding the neural substrates of reward-related decision-making with functional MRI.. Proc Natl Acad Sci U S A.

[31] Knutson B, Adams CM, Fong GW, Hommer D (2001). Anticipation of increasing monetary reward selectively recruits nucleus accumbens.. Journal of Neuroscience.

[32] McClure SM, Berns GS, Montague PR (2003). Temporal prediction errors in a passive learning task activate human striatum.. Neuron.

