# Supplementary figures and images for: Functional MRI in Awake Unrestrained Dogs
Source: PLoS One. 2012 May 11;7(5):e38027. doi: 10.1371/journal.pone.0038027 (PMC3350478; doi:10.1371/journal.pone.0038027)

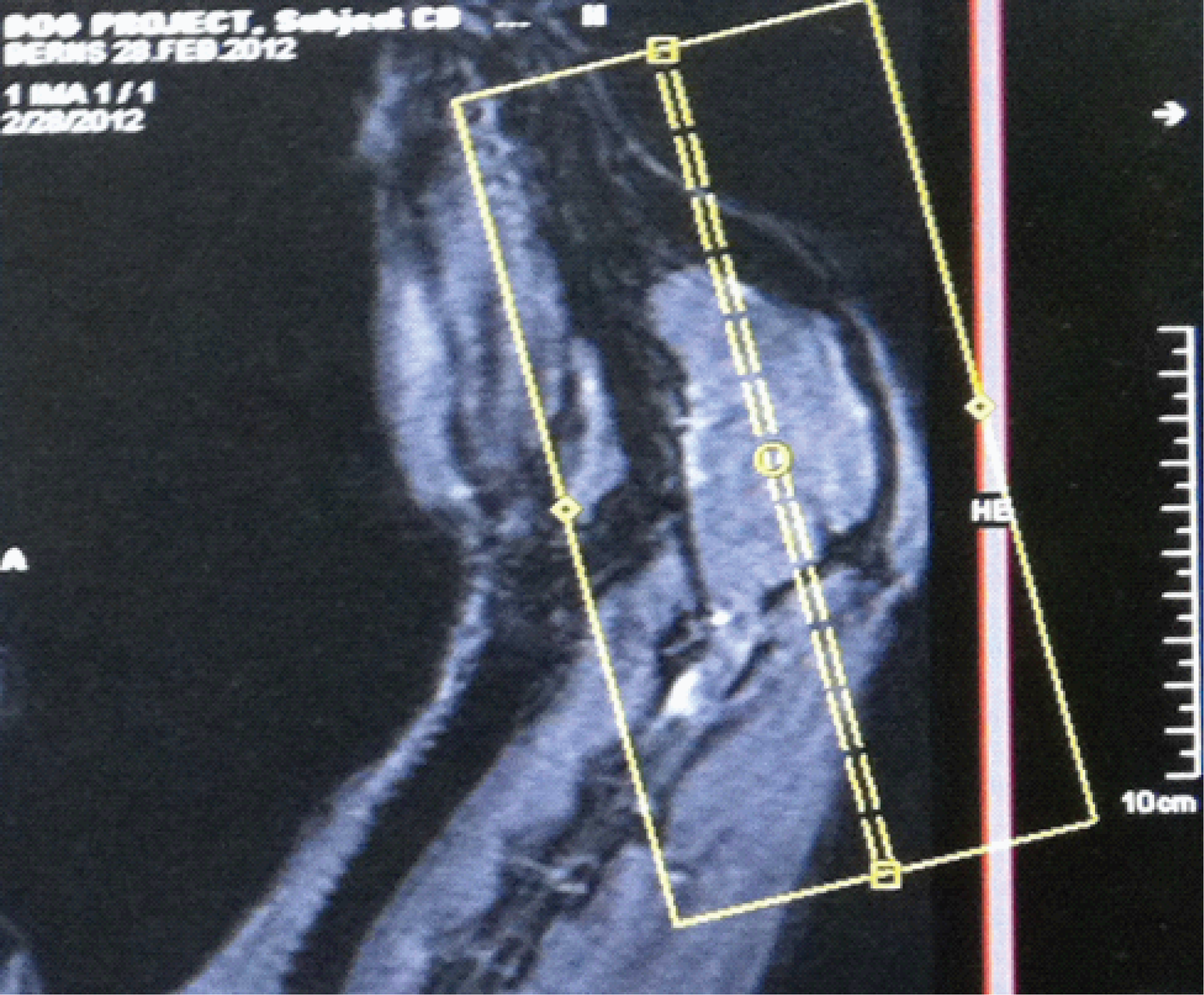

Supplement: Figure S1 — Siemens 3 T Trio console screenshot, showing field-of-view (FOV) in Callie for both functional and structural scans. The FOV was determined based on a localizer acquired prior to functional scan acquisition. Slices for the functional run were oriented dorsally to the dog's brain (similar to axial in humans). This was approximately coronal to the magnet because the dog was positioned 90° from the usual human orientation. The generous FOV, with extra slices dorsally and ventrally, allowed for different head positioning between trials. (TIF) [file pone.0038027.s001.tif]

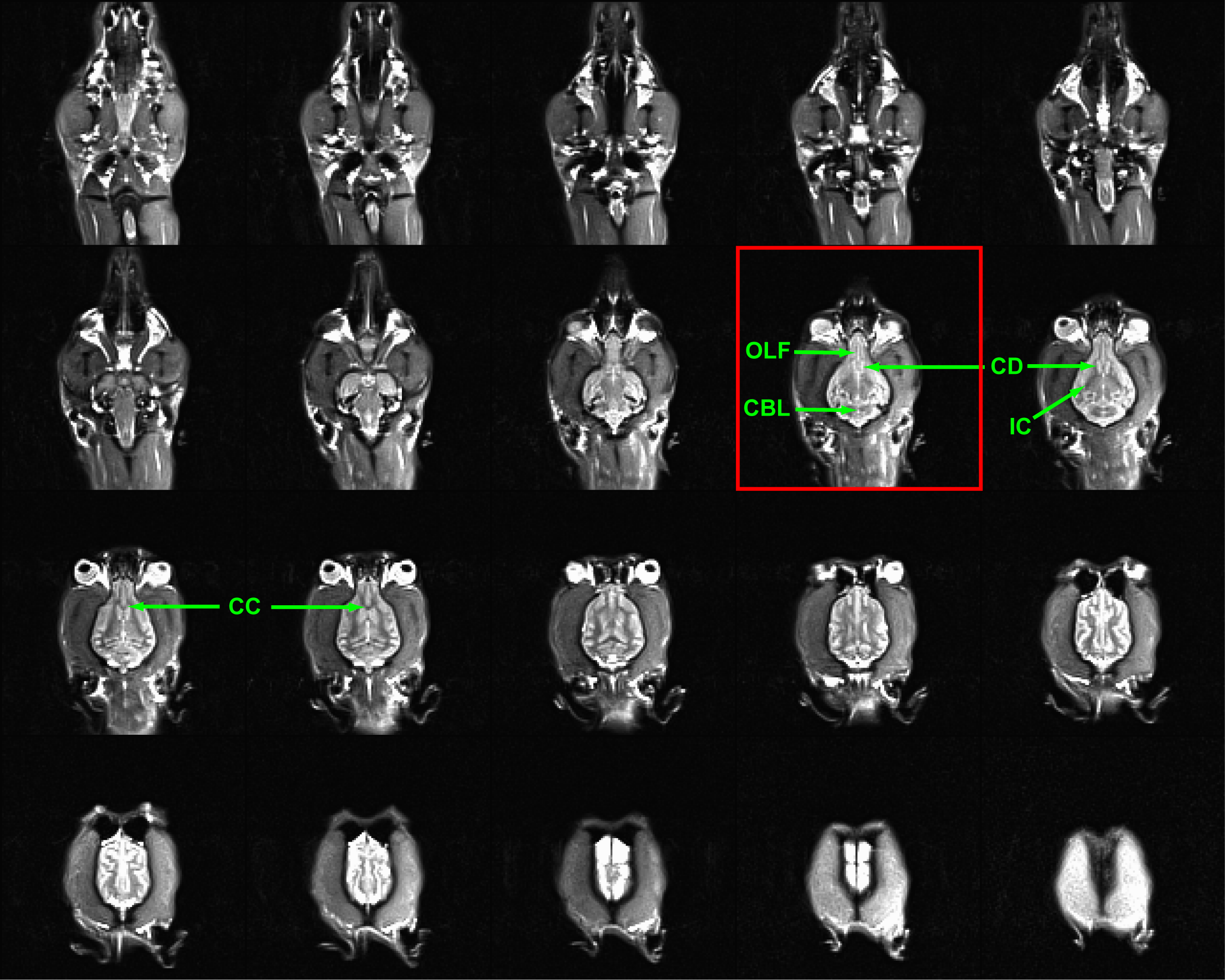

Supplement: Figure S2 — Labeled montage of Callie's T2-weighted structural image. A T2-weighted structural image was acquired after the functional runs. The image was acquired using a turbo spin-echo sequence (30 3 mm slices, TR = 3710, TE = 8.3, 26 echo trains), which was optimized to yield contrast between gray and white matter in the fastest possible time. The red outline corresponds to the slice shown in Fig. 3, where the caudate shows greater activation to the reward hand signal versus no-reward hand signal. Primary and adjacent slices are labeled with the olfactory peduncle (OLF), cerebellum (CBL), caudate (CD), internal capsule (IC), and genu of the corpus callosum (CC). (TIF) [file pone.0038027.s002.tif]

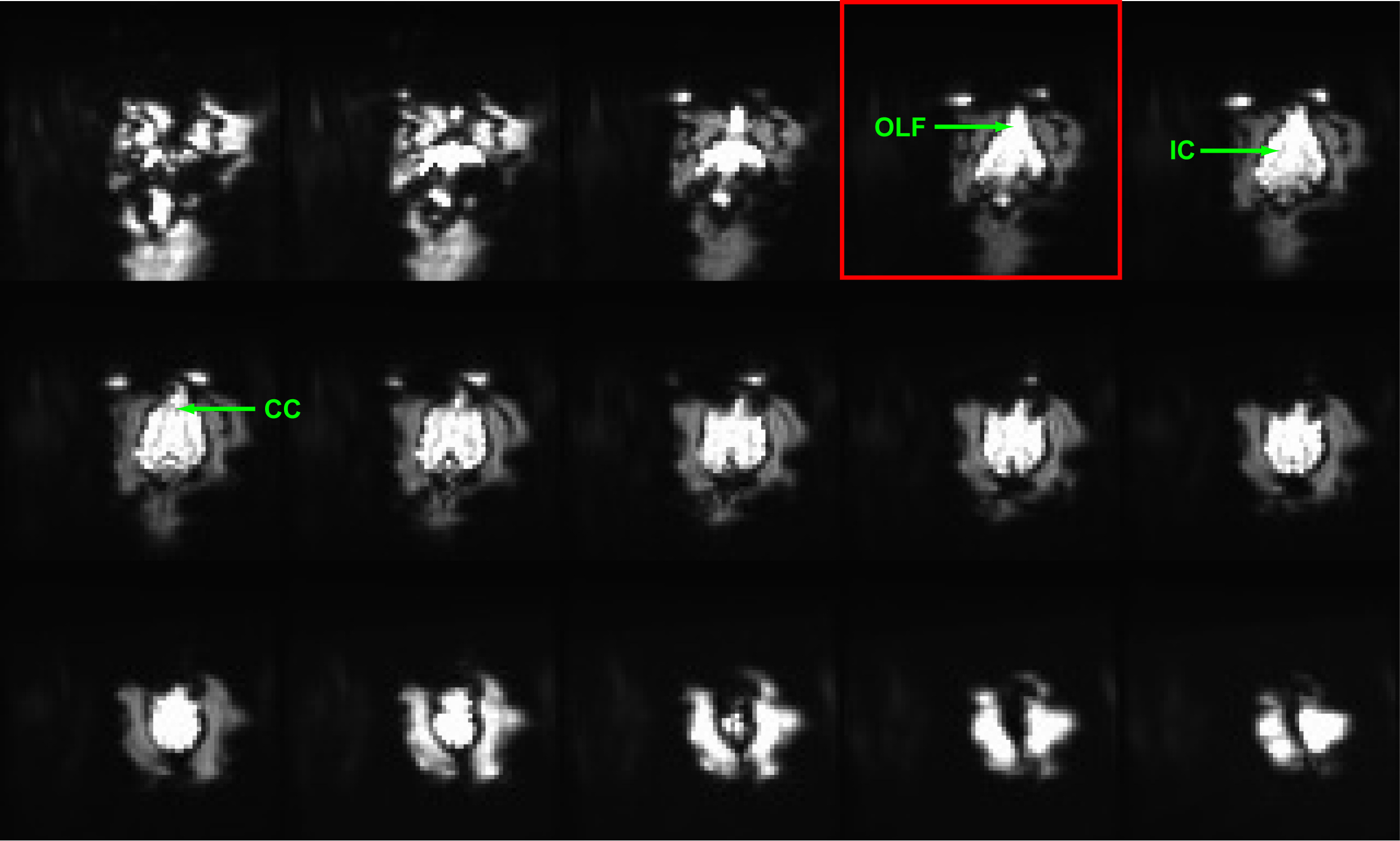

Supplement: Figure S3 — Labeled montage of Callie's mean motion-corrected EPI image. EPI images were acquired using single-shot echo-planar imaging (28 3 mm slices, 10% gap, TE = 28 ms, TR = 1610 ms, flip angle = 70°, FOV = 192 mm). The mean image across runs was calculated by taking the average of all motion-corrected EPI volumes that did not exhibit significant motion artifact. This included obvious motion artifact related to withdrawal from the radiofrequency coil, and those volumes in which the average signal changed more than 1%. The red outline corresponds to the slice shown in Fig. 3, where the caudate shows greater activation to the reward hand signal versus no-reward hand signal. Primary and adjacent slices are labeled with easily distinguishable landmarks: the olfactory peduncle (OLF), internal capsule (IC), and genu of the corpus callosum (CC). (TIF) [file pone.0038027.s003.tif]

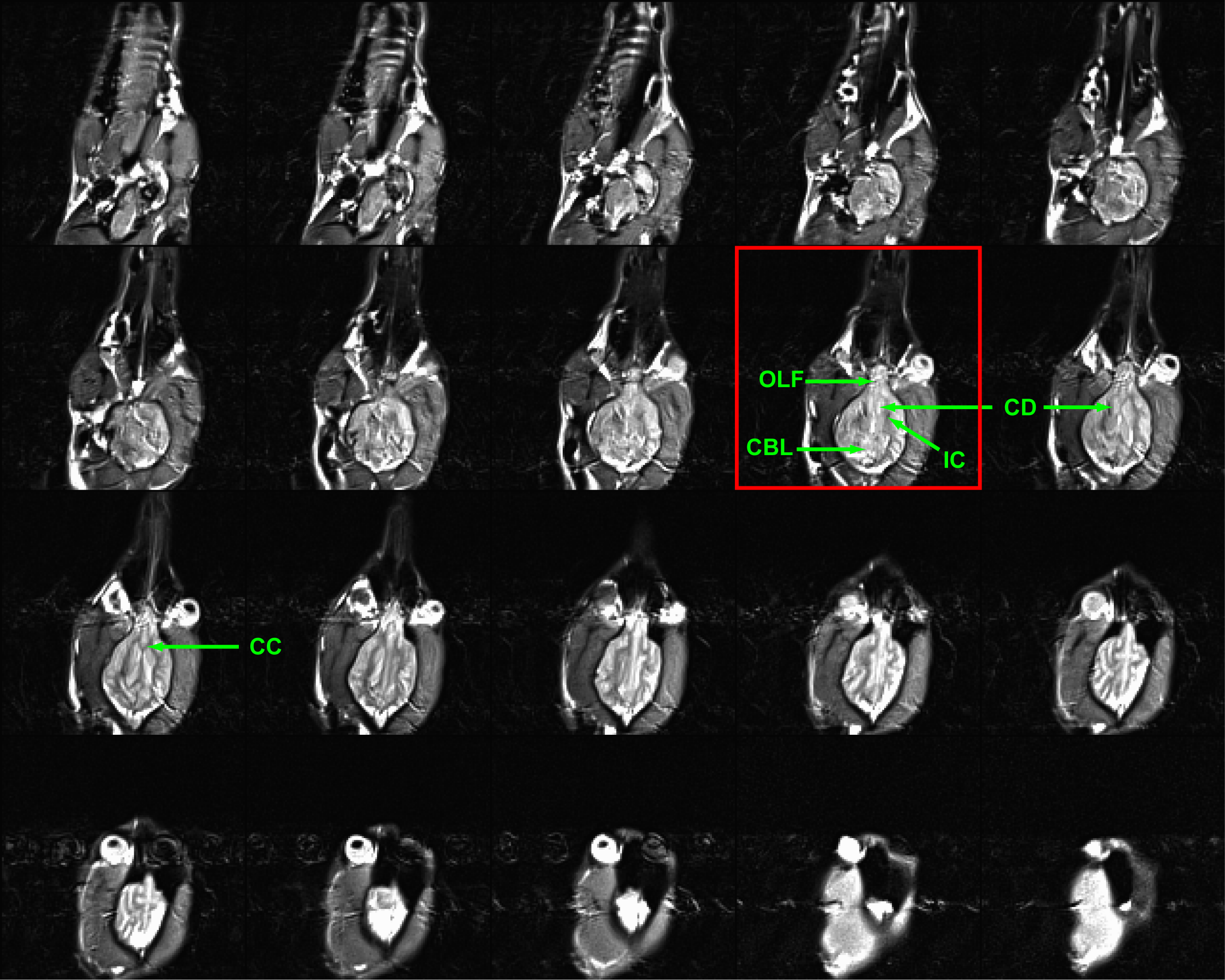

Supplement: Figure S4 — Labeled montage of McKenzie's T2-weighted structural image. A T2-weighted structural image was acquired after the functional runs. The image was acquired using a turbo spin-echo sequence (30 3 mm slices, TR = 3710, TE = 8.3, 26 echo trains), which was optimized to yield contrast between gray and white matter in the fastest possible time. The red outline corresponds to the slice shown in Fig. 3, where the caudate shows greater activation to the reward hand signal versus no-reward hand signal. Primary and adjacent slices are labeled with the olfactory peduncle (OLF), cerebellum (CBL), caudate (CD), internal capsule (IC), and genu of the corpus callosum (CC). (TIF) [file pone.0038027.s004.tif]

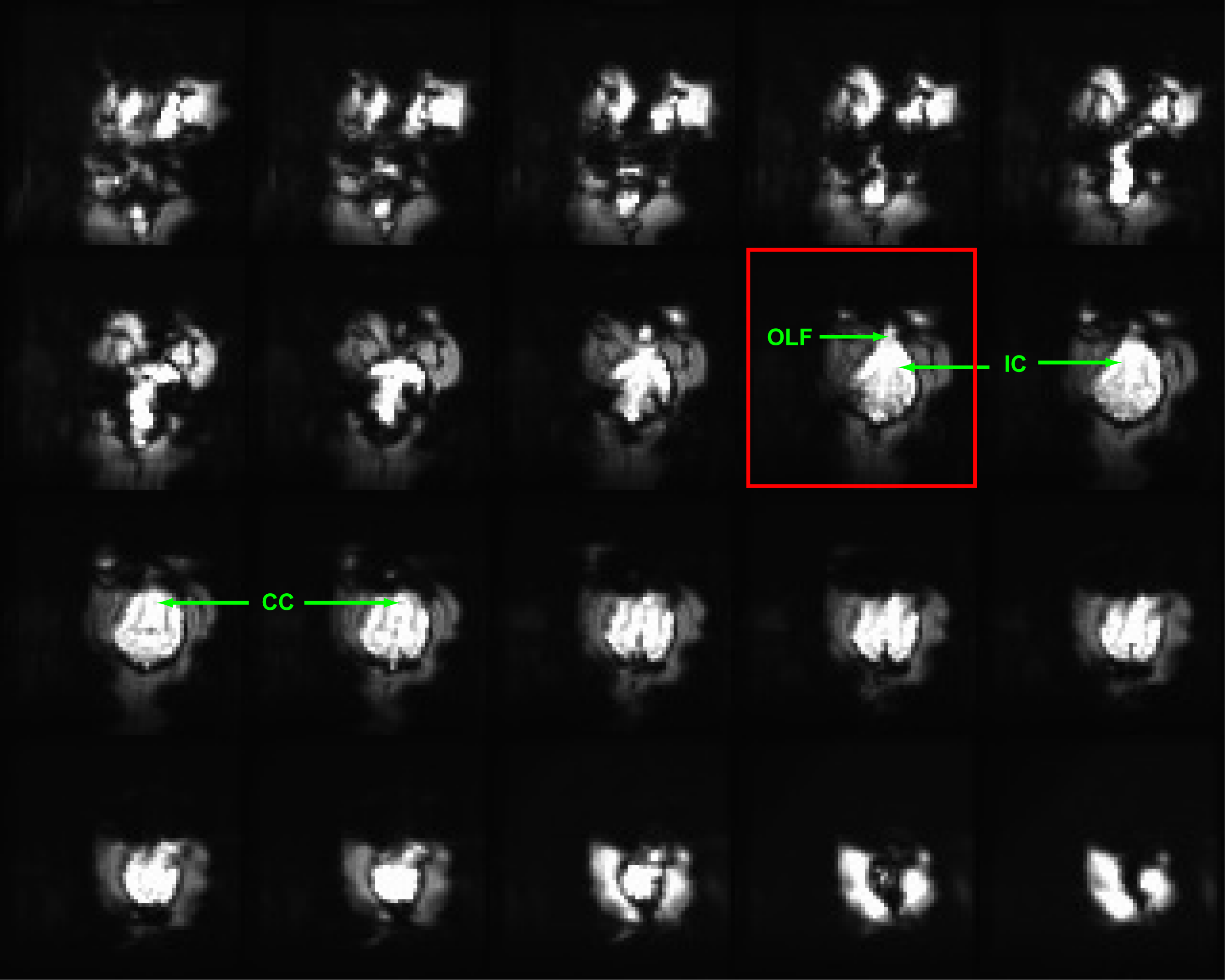

Supplement: Figure S5 — Labeled montage of McKenzie's mean motion-corrected EPI image. EPI images were acquired using single-shot echo-planar imaging (28 3 mm slices, 10% gap, TE = 28 ms, TR = 1610 ms, flip angle = 70°, FOV = 192 mm). A mean image across runs was calculated by taking the average of all motion-corrected EPI volumes that did not exhibit significant motion artifact. These included obvious motion artifact from head withdrawal from the coil, and those volumes in which the average signal changed more than 1%. The red outline corresponds to the slice shown in Fig. 3, where the caudate shows greater activation to the reward hand signal versus no-reward hand signal. Primary and adjacent slices are labeled with easily distinguishable landmarks: the olfactory peduncle (OLF), internal capsule (IC), and genu of the corpus callosum (CC). (TIF) [file pone.0038027.s005.tif]
